# Supplementary material for: Characterization of the Complete Mitochondrial Genome of Wintersweet (Chimonanthus praecox) and Comparative Analysis within Magnoliids
Source: Life (Basel). 2024 Jan 25;14(2):182. doi: 10.3390/life14020182 (PMC10890521; doi:10.3390/life14020182)
Supplement: Supplementary file 1 [file Supplementary figures.pdf › Supplementary figures.pdf]

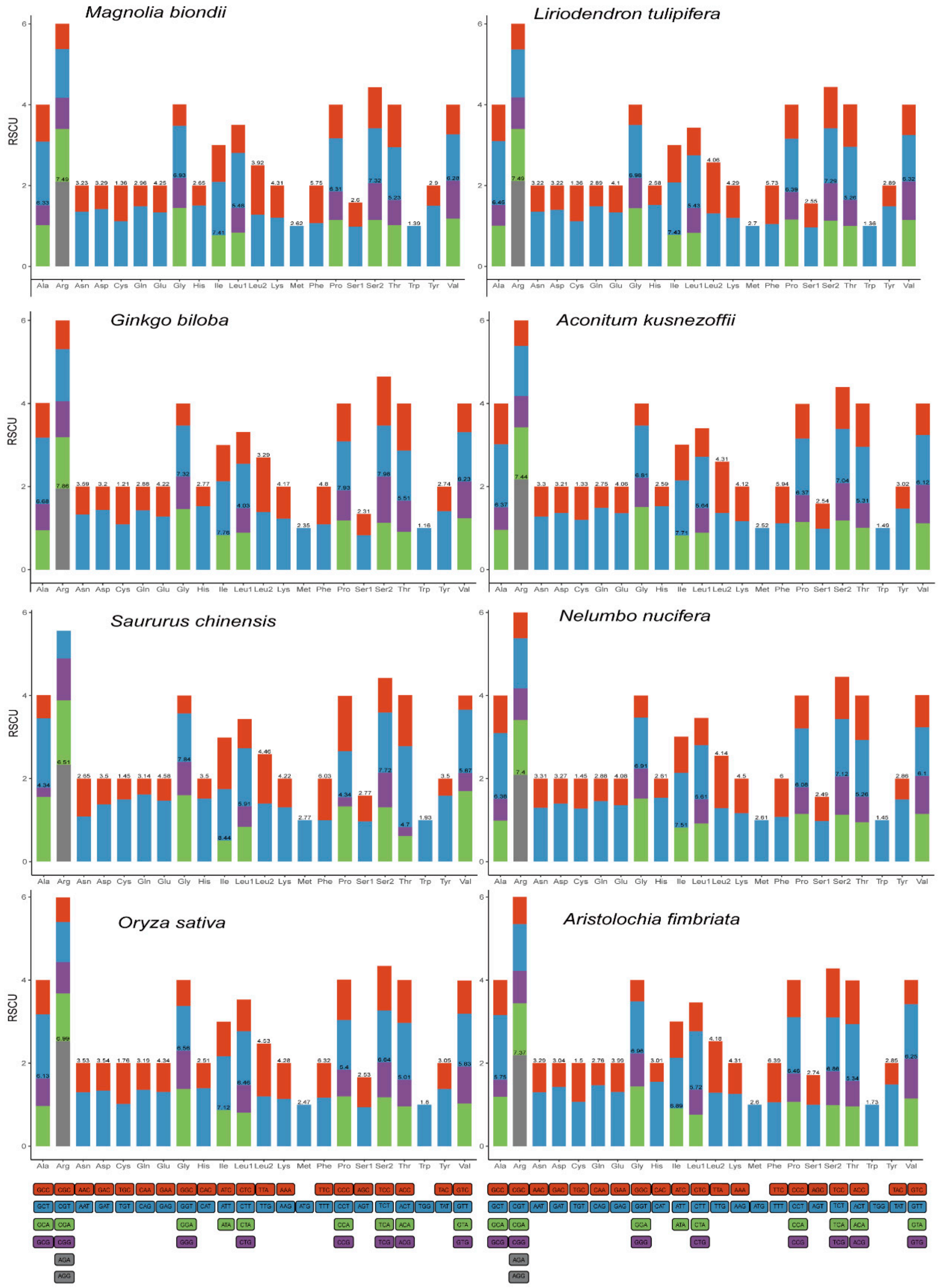

Figure S1. The codon usage analysis of protein coding genes of mitogenome of eight species. Relative synonymous codon usage (RSCU) values are plotted on the y-axis.
